# Inflammatory Cytokines Associated with Multiple Sclerosis Directly Induce Alterations of Neuronal Cytoarchitecture in Human Neurons

**DOI:** 10.1007/s11481-023-10059-w

**Published:** 2023-03-02

**Authors:** Lil Meyer-Arndt, Janis Kerkering, Tess Kuehl, Ana Gil Infante, Friedemann Paul, Kamil Sebastian Rosiewicz, Volker Siffrin, Marlen Alisch

**Affiliations:** 1 Experimental and Clinical Research Center (ECRC), Charité - Universitätsmedizin Berlin und Max Delbrück Center for Molecular Medicine in the Helmholtz Association Berlin, Lindenberger Weg 80, 13125 Berlin, Germany; 2 Department of Neurology, Charité - Universitätsmedizin Berlin, corporate member of Freie Universität Berlin, Humbolt-Universität Zu Berlin, and Berlin Institute of Health, 10117 Berlin, Germany; 3 NeuroCure Clinical Research Center, Charité - Universitätsmedizin Berlin, corporate member of Freie Universität Berlin, Humbolt-Universität Zu Berlin, and Berlin Institute of Health, 10117 Berlin, Germany

**Keywords:** Cytokine, Neuron, Stem Cell Derived, Multiple Sclerosis, Neurodegeneration

## Abstract

Multiple sclerosis (MS) is a chronic inflammatory disease of the central nervous system (CNS) coined by inflammation and neurodegeneration. The actual cause of the neurodegenerative component of the disease is however unclear. We investigated here the direct and differential effects of inflammatory mediators on human neurons. We used embryonic stem cell-derived (H9) human neuronal stem cells (hNSC) to generate neuronal cultures. Neurons were subsequently treated with tumour necrosis factor alpha (TNFα), interferon gamma (IFNγ), granulocyte–macrophage colony-stimulating factor (GM-CSF), interleukin 17A (IL-17A) and interleukin 10 (IL-10) separately or in combination. Immunofluorescence staining and quantitative polymerase chain reaction (qPCR) were used to assess cytokine receptor expression, cell integrity and transcriptomic changes upon treatment. H9-hNSC-derived neurons expressed cytokine receptors for IFNγ, TNFα, IL-10 and IL-17A. Neuronal exposure to these cytokines resulted in differential effects on neurite integrity parameters with a clear decrease for TNFα- and GM-CSF-treated neurons. The combinatorial treatment with IL-17A/IFNγ or IL-17A/TNFα induced a more pronounced effect on neurite integrity. Furthermore, combinatorial treatments with two cytokines induced several key signalling pathways, i.e. NFκB-, hedgehog and oxidative stress signalling, stronger than any of the cytokines alone. This work supports the idea of immune-neuronal crosstalk and the need to focus on the potential role of inflammatory cytokines on neuronal cytoarchitecture and function.

**Graphical Abstract:**

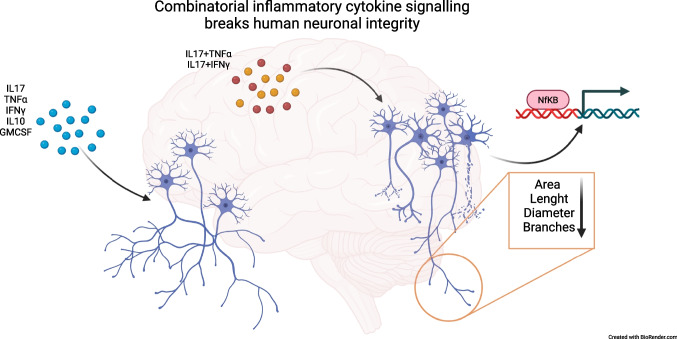

**Supplementary Information:**

The online version contains supplementary material available at 10.1007/s11481-023-10059-w.

## Background

Multiple sclerosis (MS) is a chronic inflammatory disease of the central nervous system (CNS) characterized by both inflammation and neurodegeneration, which can lead to a variety of clinical disabilities such as motor, sensory and cognitive symptoms (Reich et al. 2018; Thompson et al. 2018). Various pathobiological processes including oxidative stress, glutamate-mediated excitotoxicity and direct immune cell-mediated damage have been linked with axonal and neuronal degeneration (Nikić et al. 2011). The underlying pathobiological processes and their proportional contribution to neurodegeneration found in MS are however still heavily debated. Most of the current knowledge about neurodegeneration in MS originates from animal models of the disease (i.e., experimental autoimmune encephalomyelitis (EAE), (Friese et al. 2006). EAE mimics many clinical and neuropathological features but its comparability to the human disease is limited (Ransohoff 2012). Focusing on what is known in the human disease, in the early stages, invading lymphocytes activate the CNS-innate immune system and attract further peripheral immune cells by secreting cytokines and other pro-inflammatory mediators, which leads to the formation of demyelinating lesions (Machado-Santos et al. 2018). In later disease stages, activated microglia are the major histopathologic hallmark, which gave rise to the hypothesis that maintenance of a proinflammatory environment by persistent secretion of proinflammatory cytokines leads to neuronal damage (Jäckle et al. 2020; Zrzavy et al. 2017). Sustained cytokine production is a consistent feature throughout the disease course of MS. Several studies have shown that classical pleotropic proinflammatory cytokines such as TNFα and IFNγ, as well as more specifically employed cytokines such as GM-CSF and IL-17A, are elevated in serum or cerebrospinal fluid (CSF) of Persons with MS (PwMS, (Schofield et al. 2016; Carrieri et al. 2008; Maimone et al. 1991). A reduction was observed in IL-10 (Carrieri et al. 2008), which is known to have immune-regulatory properties. In MS, IL-10 production in blood lymphocytes inversely correlates with lesion load and clinical disability and IL-10 serum levels showed to be a risk factor for further relapses in patients with clinically isolated syndrome (Petereit et al. 2003; Wei et al. 2019). On the contrary, IL-17A secretion is associated with MRI disease activity (Hedegaard et al. 2008) and Th17 cells, the main producer of IL-17A, can disrupt the blood–brain barrier and stimulate neuroinflammation (Kebir et al. 2007). Furthermore, we have previously shown a correlation between Th17 cells in the peripheral blood and strongly neurodegenerative (T1-hypointense) lesions in MS (Bühler et al. 2017). Another cytokine associated with MS inflammation is GM-CSF, which is disproportionally frequent in MS lesions (Imitola et al. 2018) and T helper cells only producing GM-CSF have been found in the CSF of PwMS (Noster et al. 2014), (Restorick et al. 2017).

Still, the main question how the “inflammatory milieu” contributes to the disease progression is largely unanswered. Inflammatory cytokines are thought to drive immune cells to either direct cytotoxic actions or indirectly to the production of humoral effector molecules, e.g. antibodies, complement factors or apoptosis-inducing ligands. However, it has remained unclear whether inflammatory cytokines typically involved in MS disease evolution, e.g. TNFα*,* IFNγ, GM-CSF and IL-17A, can directly contribute to MS neurotoxicity. To elucidate a potential direct contribution, we established a human *in vitro* neuronal cell culture model. Human neuronal cultures were treated with recombinantly produced cytokines and cytokine combinations followed by immunofluorescence and rt-qPCR analysis.

## Material and Methods

### Cell Cultivation

We used commercially available embryonic stem cell-derived (H9) human neuronal stem cells (hNSC; Gibco) for the differentiation to neuronal cultures. hNSC were seeded on Geltrex™ (Gibco) coated wells in 2% StemPro Serum-Free Human Neural Stem Cell Culture Medium (ThermoFisher) supplemented with 2 mM GlutaMAX (Gibco), 20 ng/ml epidermal growth factor (EGF; Peprotech), 20 ng/ml basic fibroblast growth factor (ß-FGF; Peprotech) and 2% StemPro Neural Supplement (Gibco) and 1% Pen Strep (Gibco). Medium was changed every day for 5 days. After 5 days, neuronal differentiation was started by adding DMEM/F12 medium (Gibco) with 1 × B-27 supplement (Gibco), 10 ng/ml brain-derived neurotrophic factor (BDNF; Miltenyi) and 10 ng/ml neurotrophin 3 (NT3; Miltenyi) and 1% Pen Strep (Gibco). Medium was changed every other day for 14 days. After two weeks, cells were reseeded at a density of 2.6 × 10^5^ cells/cm^2^ in a Neurobasal medium (Gibco) containing 1 × B-27 supplement and 1% Pen Strep (Gibco) for neuronal maintenance. Cells were filtered before reseeding to remove any remaining stem cell conglomerates using a 100 µm filter (nylon cell strainer 100 µM, REF 352,369, Corning). Cells were cultivated another 7 days in Neurobasal medium before the experimental stage.

### Treatment with Cytokines and Inflammatory Mediators

Neuronal cell cultures were treated with IL-17, IL-10, IFNγ, TNFα, GM-CSF (concentration: 50 ng/mL; Miltenyi), l-glutamate (concentration: 250 µM; Miltenyi) or staurosporine (concentration: 0.5 µM; Selleckchem) for 24 h respectively. Concentrations of cytokines were selected based on assumed local concentrations in the CNS of PwMS as described in previous studies (Huppert et al. 2010), (Zong et al. 2016), (Ta et al. 2019), (Nasiri et al. 2020), (Neniskyte et al. 2014), (Riazi et al. 2008), (Schäbitz et al. 2007), (Vaarmann et al. 2013), (Dikmen et al. 2020). We chose the duration of treatment according to results of preliminary tests showing first signs of neuronal integrity alterations after 24 h without further changes upon prolongation of cytokine treatment.

### Immunofluorescence Staining

After 24 h of cytokine/inflammatory mediator treatment, cells were fixated (4% in paraformaldehyde (PFA) in phosphate-buffered saline (PBS)) and permeabilized (0.2% Titron X-100 in PBS; ThermoFisher Scientific) for immunofluorescence staining. Monoclonal antibodies against Class III ß-tubulin (TUBB3; 657,402, BioLegend), microtubule-associated protein 2 (MAP2; sc-74421, Santa Cruz), IL-17RA (clone G9, sc-376374, Santa Cruz), IL-10RB (clone F6, sc-271969, Santa Cruz), TNFR1 (clone H-5, sc-8436 Santa Cruz) or IFNGR1 (clone GIR 94, sc-12755, Santa Cruz) were added, and cells were incubated for 1 h at room temperature. Afterwards, matching secondary antibodies (Alexa Fluor™ 594 goat anti-mouse IgG(H + L) A11032, Alexa Fluor™ 488 goat anti-mouse IgG(H + L) A11001, Alexa Fluor™ 488 donkey anti-rabbit IgG(H + L) A21206, Alexa Fluor™ 594 goat anti-rabbit IgG(H + L) A11012, all from Invitrogen) each in a dilution of 1:1000 were applied respectively for 1 h at room temperature and DAPI (4’,6-diamidino-2-phenylindole) was used for nuclear staining. Primary antibodies were used at the following concentrations in 1% BSA/PBS upon application: mouse anti hTUBB3 (1:250), rabbit anti hMAP-2 (1:100), anti hIL-17RA (1:100), anti hIL-10RB (1:100), anti hTNFR1 (1:100), anti hIFNGR1 (1:100).

### Immunofluorescence Image Assessment

Immunofluorescence images were acquired with 20 × or 40 × magnification with a fluorescence microscope (DM6000B, Leica) and LAS X Life science software (Leica) using the same microscope settings (exposure time, gain, lamp intensity, magnification) for each experiment or test series. Images were assessed using the FilamentTracer algorithm of the commercially available IMARIS® software, which allows semi-automatic detection, tracing and measurement of neuronal cells and their processes. The software evaluates neurite features such as neurite area, neurite length, neurite diameter and neurite branches. We calculated neurite markers relative to the number of cell nuclei as ratio of a given neurite marker per nucleus.

### Intracellular Calcium (Ca^2+^) Imaging

Ca^2+^ imaging of neuronal cultures was performed on black 96-well plates (Ibidi), loaded with 1 µM Fluo-4AM (ThermoFischer Scientific) in Neurobasal Medium without phenol red (Gibco) for 15 min at 37 °C. Fluorescence imaging was performed on an inverted cell^R microscope (Olympus) within an incubation chamber at 37 °C and 5% CO_2_. Recording was conducted at 5 Hz for 5 min at 512 × 512 pixel resolution using cellSens Imaging Software. ImageJ was used for further processing.

### Cytokine Receptor Expression Analyses

Cytokine receptor expression analyses on untreated neurons were conducted using rt-qPCR in four to five independent experiments (Table 1). We used 300,000–500,000 cells per condition and experiment. RNA was isolated with Quick-RNA™ MicroPrep (Zymo Research Europe) and isolated RNA was transcribed to cDNA using High-Capacity cDNA Reverse Transcription Kit (Thermo Fisher Scientific) according to the manufacturer’s protocol. PCR was performed with SYBR® Green Fast SG qPCR Master Mix (Roboklon) according to the instructions using QuantStudio™ 5 Real-Time PCR System (Applied Biosystems).

**Table 1:** Primers used for gene expression analyses

Gene	Forward primer	Reverse primer
*TUBB3*	CCG AAG CCA GCA GTG TCT AAA CC	GCA ATA GAT TTA TTA AGT ATC CC
*MAP2*	CAT GGG TCA CAG GGC ACC TAT TC	GGT GGA GAA GGA GGC AGA TTA GCT G
*VGLUT1*	ACC TCC ATT CCA CTC ATC TC	TTT GGG TAT CCT TGA AAC TGT C
*CHAT*	ACT GGG TGT CTG AGT ACT GG	TTG GAA GCC ATT TTG ACT AT
*SLC6A*	GCC TTT TAC ATT GCT TCC TA	CCA ATT GGG TTT CAA GTA GA
*TH*	CAG TTC TCG CAG GAC ATT G	CGT CTG GTC TTG GTA GGG
*IL-17RA*	GCT TCA CCC TGT GGA ACG AAT	TAT GTG GTG CAT GTG CTC AAA
*IL-17RC*	CTG CCC TTG TGC AGT TTG G	CAG ATT CGT ACC TCA CTC CCT A
*IL-10RA*	CCT CCG TCT GTG TGG TTT GAA	CAC TGC GGT AAG GTC ATA GGA
*IL-10RB*	TCA GAA ACC TGG AGC CAT GG	AAG TGT GTT ATG ATG AGG ATG GCC
*TNFRSF1A*	TCA CCG CTT CAG AAA ACC ACC	GGT CCA CTG TGC AAG AAG AGA
*TNFRSF1B*	TGA AAC ATC AGA CTG GTG TG	TGC AAA TAT CCG TGG ATG AAG TC
*INFGR1*	AGC GAT TCC AGT ATC CTC ACT	CCA GGC TAA GCA GAA AGA GT
*INFGR2*	CTC CTC AGC ACC CGA AGA TTC	GCC GTG AAC CAT TTA CTG TCG
*GRM1*	CCA GCG ATC TTT TTG GAG GTG	TGG TGA TGG ACT GAG AAG AGG
*GRIN1*	ACG CCA TCC TAG TTA GCC ATC	GCA CGG GTA TGC GGT AGA AG
*GRIA1*	TGC TTT GTC GCA ACT CAC AGA	GGC ATA GAC TCC TTT GGA GAA C

### Signalling Pathway-Specific Target Expression Analyses

Expression of 84 genes associated with ten different signal transduction pathways in neurons incubated with IL-17, TNFα, IFNγ, IL-17/ TNFα and IL-17/IFNγ was analyzed using the Human Signal Transduction PathwayFinder™ RT^2^ Profiler™ PCR Array (PAHS-014Z, Qiagen). Total RNA of two independent experiments containing 300,000–500,000 cells per condition and experiment was isolated with Quick-RNA™ MicroPrep (Zymo Research Europe) and isolated RNA was transcribed to cDNA using the RT^2^ First Strand Kit (Qiagen) according to the manufacturer’s protocol. RT^2^ Profiler™ PCR Array was performed with RT^2^ SYBR® Green qPCR Mastermix (Qiagen) according to the instructions using QuantStudio™ 5 Real-Time PCR System (Applied Biosystems). qPCR array data were normalization against five housekeeping genes (*ACTB, B2M, HPRT1*, *GAPDH* and *RPLP0*) and relative quantification (RQ) was calculated using the ΔΔCt-Method. R was used for data presentation as a heatmap. Please note: Expression of the following genes could not be analyzed due to a lack of expression either in control samples or in all samples: *CA9* (hypoxia), *FABP1* (PPAR), *OLR1* (PPAR), *BMP2* (hedgehog), *WNT1* (hedgehog), *WNT3A* (hedgehog), *WNT6* (hedgehog), *SOCS3* (JAK-STAT), *IRF1* (JAK-STAT), *BCL2A* (NFκB), *BIRC3* (NFκB), *IFNG* (NFκB), *TNF* (NFκB), *MMP7* (Wnt), *WISP1* (Wnt).

## Results

### hNSC-Derived Neurons Exhibit Structural and Functional Properties of Mature Neurons of Mixed Phenotypes

**Fig. 1:**
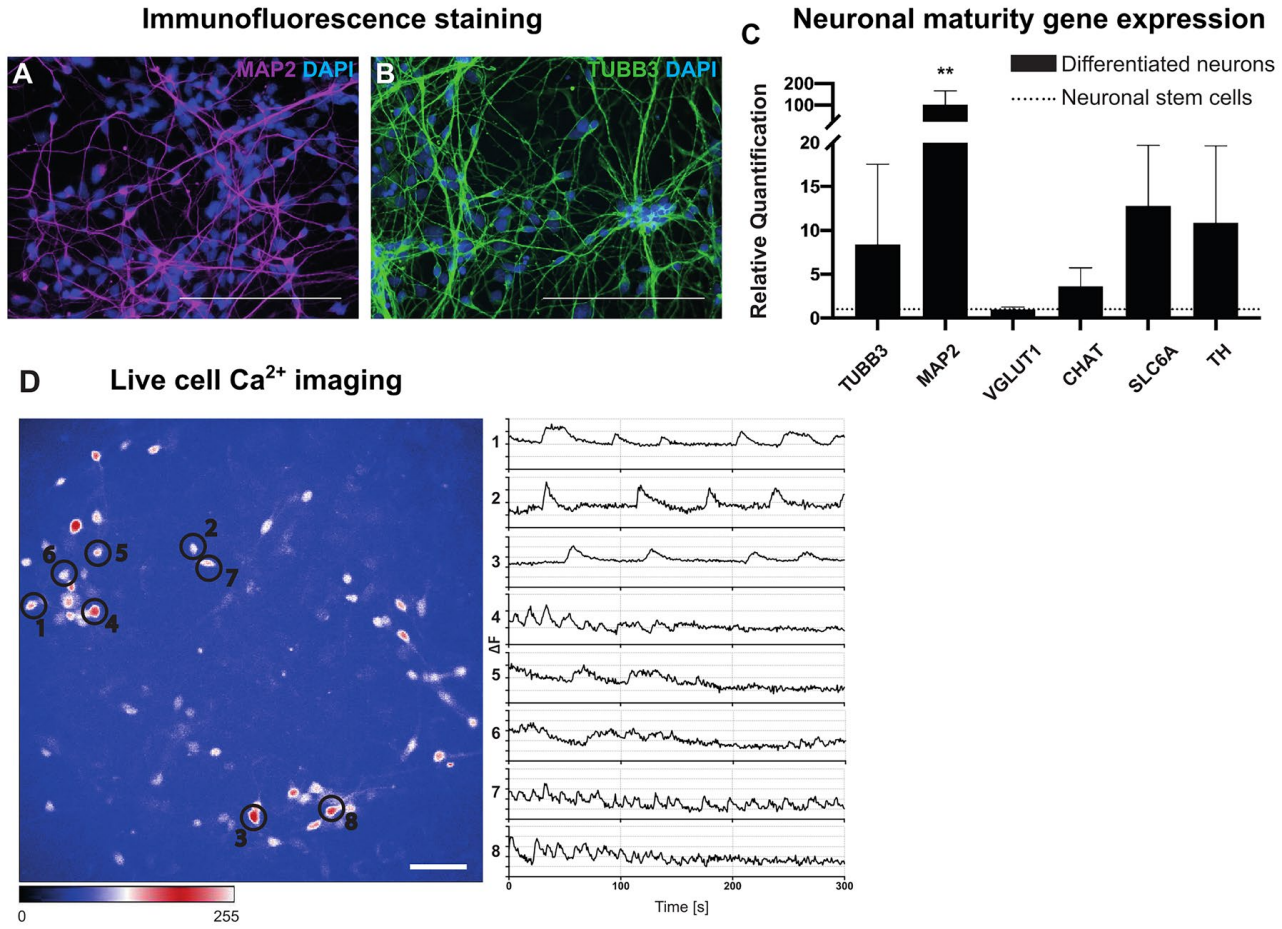
Characterization of differentiated H9-derived neurons. (**A**, **B**) Perikarya and cell processes of neurons are stained using MAP2 (A; in magenta) or TUBB3 (B; in green). Cell nuclei are stained with DAPI, scale bars 50 μm. (**C**) Expression of neuronal maturity genes (x-axis: *TUBB3, MAP2, VGLUT1, CHAT, SLC6A, TH*) in neuronal cells compared to H9-derived hNSCs as assessed using rt-qPCR (y-axis: quantification of relative changes in gene expression). Data was analysed using the Kruskal–Wallis test (*p ≤ 0.05, **p ≤ 0.01; *n* = 4 (except *VGLUT1* *n* = 3)). Outlier tests (ROUT) were performed. (**D**) Live-cell Ca^2+^ imaging was performed with Fluo-4 AM loaded neurons, which were recorded at 5–10 Hz for 5 min. Areas of spontaneous intracellular calcium transients are highlighted by black circles 1–8 to the left. Matching activity representations were calculated with ImageJ as change in fluorescence at a given time ranging from 1 to 8 from the top to the bottom (x-axis = time in milliseconds, y-axis = change in fluorescence, ΔF), scale bars 50 μm

To investigate the role of inflammatory cytokines and mediators associated with MS for neurons, we established a protocol to differentiate embryonic stem cell-derived (H9) hNSC to mixed neuronal cell cultures. We confirmed the successful neuronal differentiation by immunofluorescence staining for the neuronal maturation markers MAP2 (Fig. 1A) and TUBB3 (Fig. 1B) in line with various studies reporting neuronal specificity of these markers (Mariani et al. 2015), (Liu et al. 2007), (Caccamo et al. 1989). The results show that 94% of differentiated cells stained positive for MAP2 (Fig. 1A), which is found on both dendrites and perikarya. 89% of cells showed TUBB3-positive neurites (Fig. 1B). Furthermore, differentiated neurons demonstrated extensive neuronal network formations with branching points and neurite projections (Fig. 1A, B). As assessed with rt-qPCR, differentiated neurons expressed *MAP2, TUBB3* and further genes characteristic for different types of mature neuronal cells such as choline acetyltransferase (*CHAT*,for cholinergic neurons), solute carrier 6a (*SLC6A*; for serotonergic/GABAergic neurons), tyrosine hydroxylase (*TH*; for dopaminergic neurons) and vesicular glutamate transporter (*VGLUT1*; for glutamatergic neurons). Comparing the differentiated neurons to hNSC, neurons showed a 101-fold upregulation of *MAP2* (p ≤ 0.01), eightfold of *TUBB3* (p ≤ 0.05) as well as a fourfold up-regulation of *CHAT* (p ≤ 0.05), 13-fold of *SLC6A* (p ≤ 0.05) and 11-fold of *TH* (p ≤ 0.05) suggesting a mixed group of neuronal phenotypes, predominantly of dopaminergic and serotonergic/GABAergic neurons (Fig. 1C).

To assess cellular function, calcium (Ca^2+^) imaging by fluorescence microscopy of Fluo-4 AM loaded neuronal cultures was performed. Electrophysiological proof of spontaneous activity confirms the cell cultures’ functional maturity (Vőfély et al. 2018). Influx and redistribution of calcium ions generates intracellular signals, which are essential for neuronal functions such as synaptic plasticity and exocytosis of synaptic vesicles. Microscopic results show regular spontaneous intracellular calcium transients as expected in mature neuronal cells from different neurons (1–8), which differ in terms of their frequency and amplitude (Fig. 1D). For instance, neurons 7 and 8 show a higher firing rate than neurons 1 to 3 (Fig. 1D and Supplemental Video S1).

### Neurons Express Low but Detectable Levels of Receptors of Inflammatory Cytokines

Next, differentiated neuronal cultures were exposed to different mediators associated with neuroinflammation such as the proinflammatory cytokines TNFα, IFNγ, GM-CSF and IL-17A and the anti-inflammatory IL-10. Changes in cellular morphology and gene regulation potentially affecting cellular integrity and homeostasis were assessed using immunofluorescence staining and quantitative polymerase chain reaction (qPCR). In order to evaluate the basic potential of neurons to respond to these cytokines, we assessed the expression of cytokine receptors using immunofluorescence staining.

**Fig. 2:**
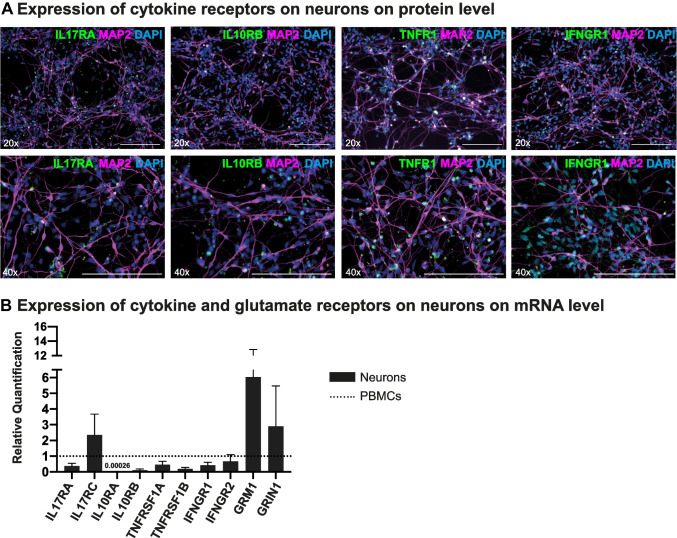
Expression of cytokine receptors on H9-derived neurons. (**A**) Neuronal cultures were stained for the cytokine receptors IL-17RA, IL-10RB, TNFR1 (α-chain) and IFNGR1 (α-chain) in green respectively, for MAP2 (in magenta) and for DAPI (in blue). Neurons demonstrated a dense expression of receptors for IFNγ and TNFα mainly localized around the perikarya. Receptors for IL-17 and IL-10 were expressed on a lower level. Scale bars 50 μm. (**B**) Expression of cytokine and inflammatory mediator receptors (x-axis) on neurons compared to PBMC as assessed with rt-qPCR (y-axis: quantification of relative changes in gene expression). All rt-qPCR were repeated four to five times on independent samples. Black bars represent neurons, dotted line PBMC. Data was analysed using the Kruskal–Wallis test, no significant results

We found that neurons expressed the receptors TNFR1 (for TNFα) and IFNGR1 (for IFNγ) on a medium level as well as IL-17RA (for IL-17A) on a low level and IL-10RB (for IL-10) on a very low level (Fig. 2A). No distinct cytokine receptor distribution patterns were associated with specific neuronal subtypes as assessed in phenotypically mixed neuronal cultures (Supplemental Fig. 1). Assessing cytokine and mediator receptor expression using rt-qPCR, we compared the cytokine receptor expression between differentiated neurons and peripheral blood mononuclear cells (PBMCs). Genes for *IL-17RA* (0.4-fold), *IL-10RA* (0.005-fold), *IL-10RB* (0.1-fold), *TNFRSF1A* (0.5-fold), *TNFRSF1B* (0.2-fold), *IFNGR1* (0.4-fold) and *IFNGR2* (0.7-fold) were expressed on a lower level and the glutamate receptors *GRM1* (6.0-fold) and *GRIN1* (2.8-fold) and *IL-17RC* (2.4-fold) on a higher level than in PBMCs (Fig. 2B). The glutamate receptor *GRIA1* was expressed on neurons but not detectable on PBMCs. To evaluate cytokine receptor expression changes upon neuronal exposure to these cytokines, we repeated the same experiment in neurons, which were treated with IL-17A, IL-10, TNFα, IFNγ or -glutamate (Supplemental Fig. 2). Neuronal cultures exposed to IFNγ showed the most distinct expression changes compared to untreated cells (i.e., upregulation of cytokine receptors investigated except for IFNGR1 and IFNGR2). By contrast, IL-17A triggered an overall cytokine receptor downregulation, most profoundly for TNFRSF1A and TNFRSF1B and GRM1.

### Inflammatory Cytokines TNFα, IFNγ, GM-CSF and IL-17A have a Distinct Effect on Neurite Morphology

**Fig. 3:**
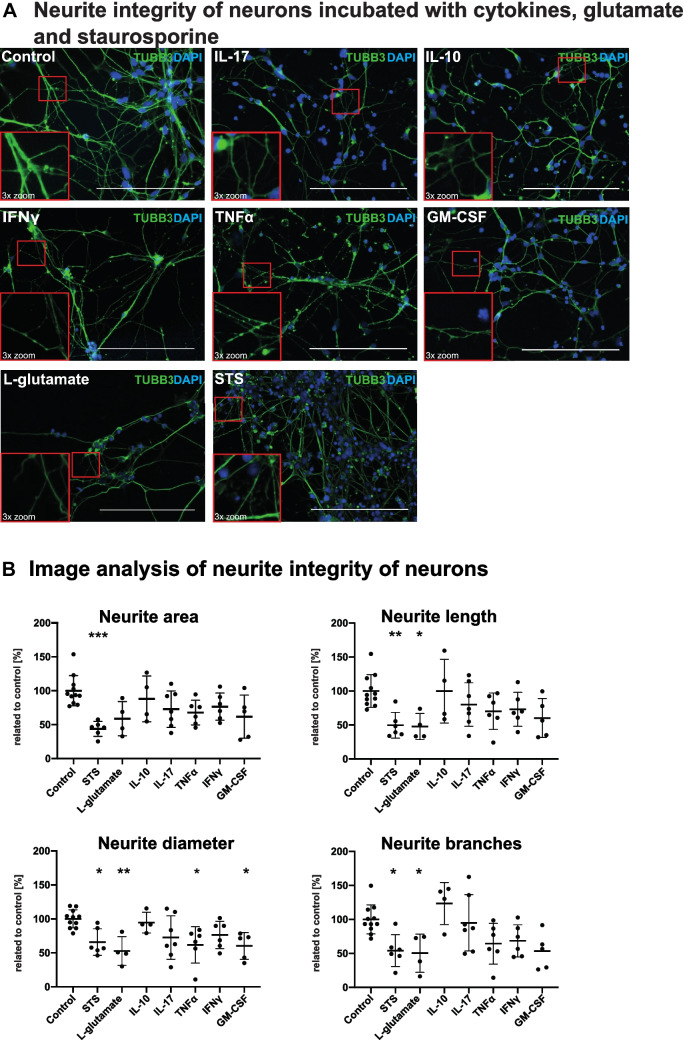
Evaluation of neurite integrity of neurons incubated with l-glutamate, staurosporine and different cytokines. (**A**) Immunofluorescence staining of the neuronal marker TUBB3 (in green) and DAPI (in blue) of neuronal cultures treated with IL-10, IL-17A, TNFα, IFNγ, GM-CSF, l-glutamate and staurosporine. Scale bars 50 μm. (**B**) Image analysis of immunofluorescence images using IMARIS®. The parameters neurite area, neurite length, neurite diameter and neurite branches are shown. X-axis displays respective cytokine treatments; y-axis shows integrity parameters per cell nucleus relative to a control in [%]. Experiments were repeated 11 × for the control condition, 6 × for staurosporine, 4 × for l-glutamate, 4 × for IL-10, 7 × for IL-17, 6 × for TNFα, 6 × for IFNγ and 5 × for GM-CSF. Dots in graphs show mean values of four images per experiment and experimental condition (IL-10 *n* = 4, IL-17A *n* = 7, TNFα *n* = 6, IFNγ *n* = 6, GM-CSF *n* = 5, l-glutamate *n* = 5, staurosporine *n* = 6). Data was analysed using the Kruskal–Wallis test (*p ≤ 0.05, **p ≤ 0.01, **p ≤ 0.001). Outlier tests (ROUT) were performed on all experiments

Next, we assessed the cellular integrity after incubation of H9-hNSC-derived neuronal cultures with cytokines and neurotoxic substances, e.g. staurosporine (apoptosis-inducing protein kinase inhibitor) or l-glutamate (excitotoxic transmitter). We compared the effect of the classical proinflammatory mediators TNFα, IFNγ and GM-CSF to IL-17A and anti-inflammatory IL-10 on neuronal cell integrity using immunofluorescence staining. For this purpose, we incubated neuronal cultures with the cytokines/mediators (concentrations: 50 ng/mL for IL-17A, IL-10, IFNγ, TNFα, GM-CSF; 250 µM for l-glutamate; 0.5 µM for staurosporine) for 24 h. After incubation, we performed immunofluorescence staining for TUBB3 as essential protein of the neuronal cytoskeleton. We analysed neurite integrity using the FilamentTracer algorithm of the commercially available IMARIS® software. We quantified neurite area, neurite length, neurite diameter and number of neurite branches per cell nucleus in a blinded analysis (Fig. 3B). Staurosporine or l-glutamate treated samples served as positive controls. Exposure to staurosporine led to a significant reduction of neuronal processes (Fig. 3A) with all neurite integrity parameters being significantly impacted (Fig. 3B). L-glutamate showed a similar effect for all parameters (Fig. 3B). The impact of TNFα, IFNγ and GM-CSF on neurite morphology and branching was unlike the one observed in the neurotoxic controls. Upon treatment with each of the three pro-inflammatory cytokines, neurons display a “pearl-on-string” morphology as potential sign of disturbed axonal trafficking and a reduced branching (Fig. 3A). TNFα and GM-CSF caused a decrease of neurite integrity parameters though statistically significant only for the reduction of neurite diameter per cell nucleus by TNFα (61.8%; p ≤ 0.05) and neurite diameter and branches per nucleus by GM-CSF (60.2%; p ≤ 0.05; 53.4%; p ≤ 0.05; Fig. 3B). IL-17A showed a similarly decreasing, albeit non-significant effect on most of the neurite integrity parameters (Fig. 3B). Interestingly, neurons exposed to IL-17A display an overall thinning of neurite processes as demonstrated in Fig. 3A. In line with the findings of very low receptor expression, we could not find any characteristic morphologic alterations (Fig. 3A) or significant changes of neurite parameters after IL-10 treatment (Fig. 3B).

### Combinatorial Treatment with IL-17A/IFNγ and IL-17A/TNFα Leads to more Pronounced Effects on Neuronal Integrity than with IL-17A Alone

**Fig. 4:**
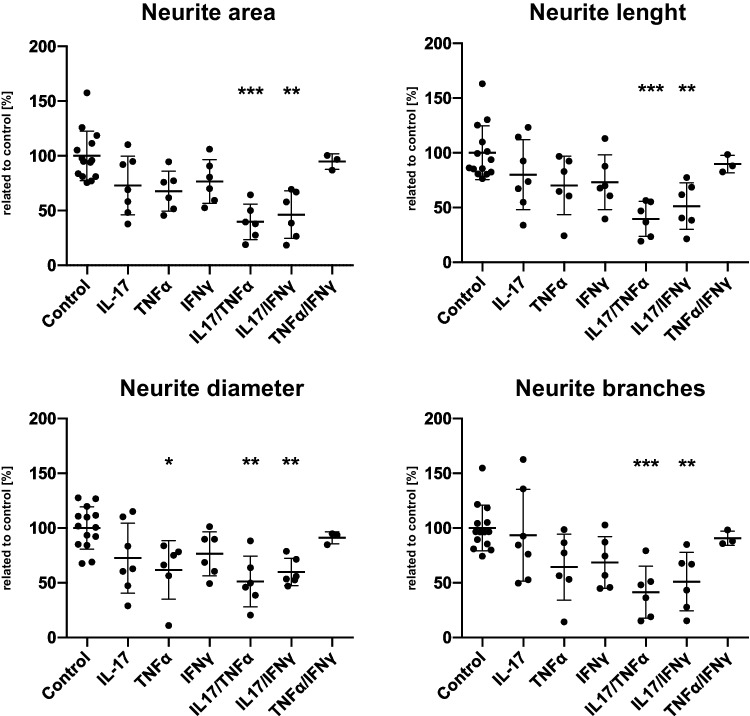
Impact of combinatorial cytokine incubation on neurite integrity. Image analysis of immunofluorescence staining for the neuronal marker TUBB3. X-axis displays respective combinatorial cytokine treatments; y-axis displays integrity markers per cell nucleus. Dots in graphs show mean values of four images per experiment and experimental condition (IL-17A *n* = 7, TNFα *n* = 6, IFNγ *n* = 6, IL-17/TNFα *n* = 6, IL-17/IFNγ *n* = 6, TNFα/IFNγ *n* = 3). Data was analysed using the Kruskal–Wallis test (*p ≤ 0.05, **p ≤ 0.01, **p ≤ 0.001). Outlier tests (ROUT) were performed on all experiments

As a release of proinflammatory mediators is usually an orchestrated process involving several of these mediators, we were interested in whether simultaneous neuronal treatment with IL-17A and one of the well-described proinflammatory cytokines TNFα and IFNγ may synergistically enhance the effect of IL-17A on neurite integrity. Neuronal cultures were incubated with either IL-17A/TNFα or IL-17A/IFNγ (concentrations: 50 ng/mL for IL-17A, TNFα and IFNγ respectively) for 24 h. We additionally exposed neurons to a combination of TNFα/IFNγ for comparison. Our results show that combinatorial incubation with IL-17A had an amplified impact on neuronal integrity markers extending distinctly beyond the effect of IL-17A, IFNγ or TNFα alone. Both combinations including IL-17A triggered a significant decrease of all cell integrity markers (p ≤ 0.01—p ≤ 0.001; Fig. 4). In line with the results of single treatments, this decrease was slightly more pronounced in samples treated with IL-17A in combination with TNFα than with IFNγ. We found the largest impact to be on neurite area (IL-17A/TNFα ratio 39.8%; IL-17A/IFNγ ratio 46.3%) and neurite length (IL-17A/TNFα ratio 39.7%; IL-17A/IFNγ ratio 51.3%; Fig. 4). By contrast, a combined treatment of neurons with TNFα/IFNγ showed a less deteriorating and non-significant effect on cell integrity parameters than either of these mediators in combination with IL-17A resembling single cytokine exposure.

### NFκB Pathway Induction is Amplified by Combinatorial Neuronal Exposure to IL-17A/IFNγ, IL-17A/TNFα or TNFα/IFNγ

**Fig. 5:**
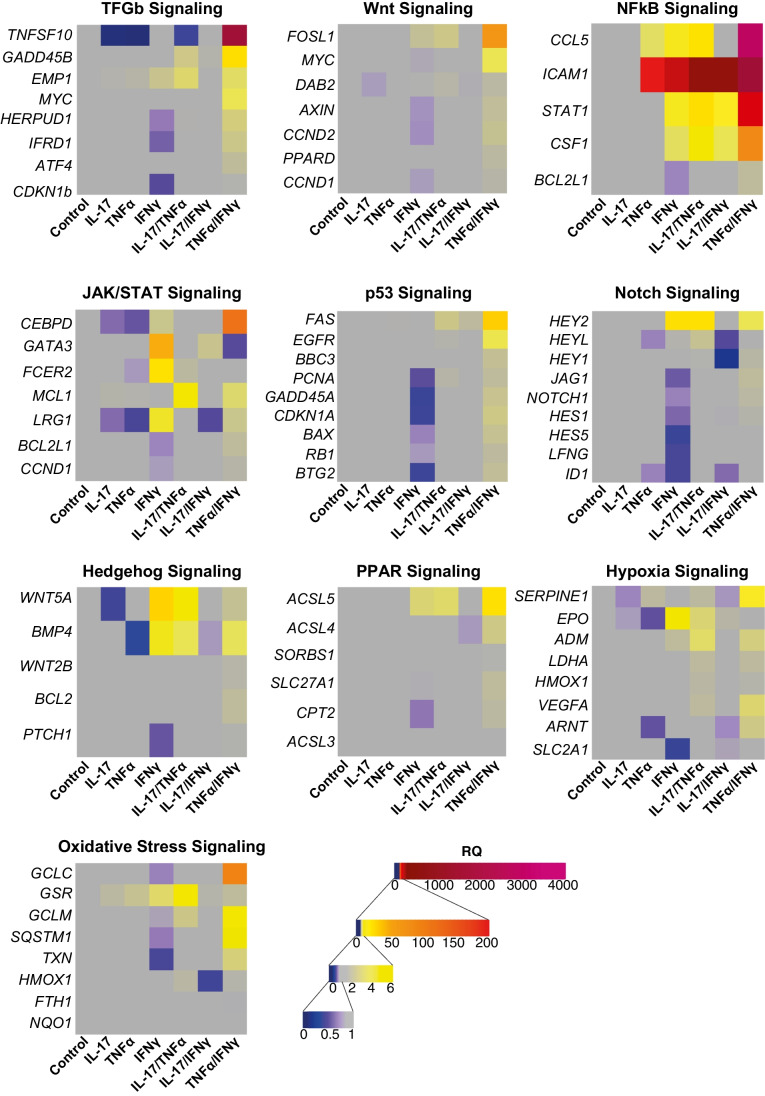
Signalling pathway alterations in neuronal cells upon mediator treatment. Regulation of signalling pathway-specific target genes in neuronal cells after 24 h of treatment with individual proinflammatory mediators (x-axis: IL-17A, TNFα, IFNγ and combination of IL-17A/TNFα, IL-17A/IFNγ and TNFα/IFNγ (concentration: 50 ng/mL)) in comparison to untreated cells as assessed with rt-qPCR arrays (y-axis: respective pathway-specific target genes). Colours quantify RQ in gene expression. Rt-qPCR arrays were performed twice with independent samples (pooled data shown)

To investigate how pro-inflammatory mediators impact signalling pathways in hNSC-derived neurons, we analysed expression regulation of target genes associated with ten different signalling pathways upon neuronal treatment with single or combinatory inflammatory mediators for 24 h using a commercially available rt-qPCR array (Fig. 5). Here, we found that TNFα and IFNγ showed the most pronounced impact on the NFκB pathway with up-regulation for *ICAM1* (encodes for a transmembrane intercellular adhesion glycoprotein; RQ 54.73 (TNFα), 105.08 (IFNγ)), *CCL5* (chemotactic cytokine; RQ 3.44 (TNFα), 6.60 (IFNγ)), *CSF1* (cytokine which induces differentiation into macrophages; RQ 1.19 (TNFα), 3.50 (IFNγ)) and *STAT1* (cytokine signalling relevant transcription factor; 6.74 (IFNγ)). This target gene up-regulation was further amplified upon combinatorial treatment with IL-17A particularly in combination with TNFα or combination of TNFα and IFNγ: *ICAM1* RQ 162.47 (IL-17/TNFα), RQ 2066.08 (TNFα/IFNγ), *CCL5* RQ 9.26 (IL-17/TNFα), RQ 3442.26 (TNFα/IFNγ), *CSF1* RQ 5.48 (IL-17/TNFα), RQ 21.97 (TNFα/IFNγ) and *STAT1* RQ 9.96 (IL-17/TNFα), RQ 84.08 (TNFα/IFNγ). Interestingly, IL-17A alone did not lead to an increase in target gene expression of the NFκB pathway or any other pathway investigated. However, combinatorial exposure of neurons to IL-17A and TNFα caused a target gene up-regulation that exceeded single treatments including those of TNFα alone. Furthermore, other signalling pathways were also activated by IL-17/TNFα or TNFα/IFNγ: oxidative stress (glutathione reductase (*GSR*) RQ 6.82 (IL-17/TNFα), RQ 1.99 (TNFα/IFNγ)), hypoxia (erythropoietin (*EPO*) RQ 3.0 (IL-17/TNFα), vascular endothelial growth factor A (VEGFA) RQ 3.11 (TNFα/IFNγ)), hedgehog (growth factors *WNT5A* RQ 5.53 (IL-17/TNFα), RQ 2.22 (TNFα/IFNγ) and *BMP4* RQ 3.85 (IL-17/TNFα), RQ 3.64 (TNFα/IFNγ)) and peroxisome proliferator-activated receptor (PPAR; *ACSL5* RQ 3.27 (IL-17/TNFα), RQ 9.51 (TNFα/IFNγ)). These regulation patterns were comparable to those of single IFNγ (oxidative stress (glutathione reductase (*GSR*) RQ 3.28), hypoxia (erythropoietin (*EPO*) RQ 4.9), hedgehog (*WNT5A* RQ 11.59, *BMP4* RQ 4.75), peroxisome proliferator-activated receptor (PPAR; enzyme *ACSL5* RQ 3.01)) and were not triggered by single IL-17A or TNFα exposure alone. Comparable to TNFα, IL-17A alone did not up-regulate signalling pathway target genes except for gsr (RQ 1.87) but rather decreased expression of target genes involved in the TGFb (RQ 0.08), JAK/STAT (*lRG1* RQ 0.57, *CEBPD* RQ 0.57), hedgehog (*WNT5A* RQ 0.36) and hypoxia (*SERPINE1* RQ 0.64, *EPO* RQ 0.70) pathways.

## Discussion

In this study, we show that proinflammatory cytokines frequently found and cited in the pathophysiology of MS have direct effects on neurons and their cellular integrity in a human *in-vitro* neuronal cell culture model. We identified cytokine receptor expression on mature mixed neuronal cultures using immunofluorescence staining and qPCR. Treatment with the respective cytokines and in particular with cytokine combinations had distinct effects on neuronal morphology and intracellular signalling events. This is to our best knowledge the first study on direct effects of inflammatory cytokines and cytokine combinations on human neuronal cytoarchitecture. All data presented were collected from an embryonic stem cell derived hNSC cell line with no reported disease. Therefore, these findings might be relevant not only for MS, but for any CNS disease involving the investigated cytokines.

The common idea of MS pathogenesis consists of a persistent pathological immune response evoked by lymphocytes entering the CNS. In this paradigm, peripheral immune cells enter the CNS, induce demyelination and activate CNS-endogenous immune cells, which in the long-run leads to neuronal and axonal damage. The mechanisms leading from inflammation and demyelination to axonal and neuronal damage have, however, remained unclear. Here, we evaluated the role of cytokines and cytokine combinations as part of an immune-neuronal crosstalk. We show that mature human neuronal cultures express cytokine receptors already in the steady state, which is furthermore modulated upon exposure to these pro-inflammatory cytokines. Among these cytokines is the pleiotropic TNFα. TNFα signalling is multifaceted and complex, i.e., depending on temporality and location of its expression, and TNFα has been reported to have both beneficial and detrimental effects in a context-dependent manner (Bruce et al. 1996; Downen et al. 1999). Two high-affinity cell surface receptors with distinct regulatory effects named TNFR1 and TNFR2 recognize TNFα, which exists in a soluble and a transmembrane form. While the transmembrane form is considered to mediate rather beneficial effects via TNFR2, the soluble cytokine triggers pro-inflammatory pathways through TNFR1 (Chen and Goeddel 2002), which includes a death domain (Yang et al. 2018; Sedger and McDermott 2014). A genome wide association study has identified an MS risk single nucleotide polymorphism (SNP), which was associated with increased levels of soluble TNFR1 (Gregory et al. 2012). In line with this finding, TNFα inhibitors (mimicking the effect of increased levels of soluble TNFR1) were found to exacerbate the course of MS (Pegoretti et al. 2018). In our study, *TNFR1* and *TNFR2* (although in smaller quantities) were both expressed on mixed neuronal cultures. Incubation with TNFα resulted in distinct structural alterations of neuronal processes presenting a prominent beading. This effect of neurite morphology changes was levered by the cytokine IL-17A, which is relevant in context of EAE and MS. The IL-17 cytokine family, which consists of the six cytokines IL-17A to IL-17F, binds to a receptor complex formed by IL-17RA and IL-17RC (Yao et al. 1995). IL-17RA shows its highest expression in hematopoietic cells but is also expressed in non-hematopoietic epithelial and mesenchymal tissues (Ishigame et al. 2009; Kuestner et al. 2007). Our findings demonstrate that hNSC-derived neurons expressed IL-17RA (both epitope and mRNA) although to a lesser extent than immune cells. IL-17 signal transduction is well understood in cells of non-hematopoietic origin such as keratinocytes and in colonic epithelial cells. IL-17 upregulates chemokines and metalloproteases, which stimulate neutrophile recruitment and IL-17 blocking agents have been successfully used to treat psoriasis (Liang et al. 2006; Nograles et al. 2008; Lee et al. 2008; Ly et al. 2019) and also MS (Havrdová et al. 2016). Nevertheless, little is known about IL-17 signaling in the CNS. A murine EAE study showed that IL-17RA is constitutively expressed in murine astrocytes and microglia and its expression is upregulated in a proinflammatory environment (Das Sarma et al. 2009). Murine neuronal cells in the dorsal root ganglia, the spinal cord and the cortex express IL-17 receptors in-vitro as previously demonstrated (Luo et al. 2019; Segond von Banchet et al. 2013; Wang et al. 2009). IL-17 is produced by innate and adaptive immune cells and in particular by Th17 cells, which are considered crucial for chronic inflammatory and autoimmune pathologies such as MS (Korn et al. 2009). Initially, Th1 cells were considered major drivers of MS disease and a recent study found the extent of their contribution to be related to MS stage (Arellano et al. 2017). The main effector cytokine of Th1 cells is IFNγ, which binds to IFNGR1, the ligand binding chain of the IFNγ receptor. Most cells including neurons express IFNγ receptors (Bach et al. 2003). In line with our findings, Th1 and Th17 cells mediate development of EAE and Th17 cells were formerly found to induce functional damage in human neurons pointing towards an important role of these effector T cells for direct neuronal damage, which is classically attributed to cytotoxic T cells (Siffrin et al. 2010), (Loos et al. 2020).

IL-10 is considered an antagonist to these pro-inflammatory cytokines exerting immunosuppressive functions and dysregulation or deficiency is often associated with autoimmune diseases and chronic infections (Engelhardt and Grimbacher 2014; Couper et al. 2008). A large variety of immune cells produce IL-10 (Moore et al. 2003). The heterodimeric receptor complex for IL-10 consists of an alpha (IL-10RA) and a beta subunit (IL-10RB, (Yoon et al. 2010). In our human neuronal model, IL-10RB is only lowly expressed. This is in line with previous studies showing that IL-10 receptors are mainly expressed on hematopoietic cell lines but they were also found on non-hematopoietic cells such as neurons in animal models (Chen et al. 2016), (Zhou et al. 2009).

Neurotoxic effects of cytokines and mediators such as TNFα, IFNγ, GM-CSF and l-glutamate on neuronal cell integrity have been extensively investigated, mainly in animal models. In this study, we compared these known pro-inflammatory substrates to the MS-relevant cytokines IL-17A and IL-10. In human neurons, IFNγ indirectly triggered neurotoxic effects mediated by astrocytes and induced direct neurotoxic effects visualized as neurite bead formation (Mizuno et al. 2008). Direct neurodegenerative effects have also been reported by TNFα in mice through silencing of survival signals (Zhao et al. 2001; Takeuchi et al. 2006). This is in line with our findings on neuronal exposure to TNFα and IFNγ, which demonstrate a reduction in neurite integrity including branching (significant for TNFα), diameter, area and length and characteristic morphology alterations suggesting disruption of cytoarchitecture. GM-CSF classically induces cellular proliferation and differentiation and is therefore considered to promote pathogenic processes in autoimmune diseases that rely on cellular mechanisms (Lotfi et al. 2019). Consistently in the EAE model, GM-CSF promotes migration of monocytes through the BBB and induces proliferation and activation of microglia, which in turn secrete pro-inflammatory cytokines to maintain the inflammatory environment (Dikmen et al. 2020; Spath et al. 2017; Aram et al. 2019). Directly harmful effects on CNS cells have not been shown to date. We here demonstrate that GM-CSF has similar effects on neuronal integrity as seen in TNFα and IFNγ with a significant decrease of neurite branching. IL-17 was found to be the highest-ranking gene expressed in autopsy samples of PwMS (Lock et al. 2002). In mice, suppression of IL-17 slowed down EAE progression (Langrish et al. 2005; Waisman et al. 2015; Tzartos et al. 2008). In our experiments, exposing hNSC-derived neurite length and neurite diameter as compared to typical pro-inflammatory mediators. This is in line with findings by (Paintlia et al. 2011) who showed that IL-17 exacerbated oligodendrocyte loss in rats in vitro and (Kang et al. 2013) who demonstrated that IL-17 inhibits murine CNS cell maturation. In our hNSC-derived neuronal cell line, the damaging effect of IL-17A was increased in combination with IFNγ and particularly with TNFα surpassing the effects of TNFα and IFNγ alone. Interestingly, the combination of TNFα/IFNγ – although strongly inducing NFκB-associated genes – did not induce phenotype alterations of neurites comparable to the IL-17A combinations. This finding suggests that IL-17A has a distinct effect on neurons and is essential for the damaging morphology alterations observed. Other research groups have shown similar synergistic effects of IL-17 and TNFα in autoimmune liver disease and on oligodendrocytes (Paintlia et al. 2011; Beringer et al. 2018). The combinatorial effect of IL-17A/TNFα or IL-17A/IFNγ may be explained by harnessing cooperating signalling pathways. We thus studied cytokine-mediated expression regulation of multiple target genes associated with ten different signalling pathways in our neuronal cell model. Here, we found the most pronounced up-regulation of target genes assigned to the NFκB pathway. NFκB is a transcription factor essential for inflammatory responses and in this function targets genes involved in cell proliferation, cytokine release and apoptosis (Taniguchi and Karin 2018). Consistent with our findings on neurite integrity alterations, NFκB-associated gene up-regulation was again amplified upon neuronal exposure to combinatorial cytokines and in particular to IL-17A/TNFα. In contrast, IL-17A or TNFα alone (except ICAM1 and CCL5 by TNFα) did not lead to up-regulation of target genes investigated in this array. This is different to the effects of IL-17A and IFNγ in which case a combination did not increase up-regulation. Highlighting the synergistic effect of IL-17A and TNFα, we observed further IL-17A/TNFα-mediated target gene up-regulation for the hedgehog pathway, the PPAR pathway and oxidative stress and hypoxia pathways. Combination of TNFα and IFNγ showed a pronounced amplification of genes in particular in the NFκB pathway, however, also effects distinct to the IL-17A combinations in other pathways, e.g. oxidative stress signalling or hedgehog signalling. The differential effect on neurite morphology and signalling pathways warrants further investigation. The PPAR pathway is involved in energy homeostasis, lipid and glucose metabolism (Tyagi et al. 2011) and promotes anti-inflammatory neuroprotective mechanisms after brain injury (Victor et al. 2006; Villapol et al. 2015). The hedgehog pathway is classically affiliated with embryonic development and cell repair (Briscoe and Thérond 2013; Lopez-Bergami and Barbero 2020). Activation of hedgehog pathways induced by acute brain injury has previously been shown in other studies (Allahyari et al. 2019), (Wu et al. 2020) and underlines the potentially damaging effect of neuronal exposure to cytokines as demonstrated in our findings. Cytokines may reach very high local concentrations when cells directly interact. Inversely, more remote inflammatory processes can lead to lower exposure. Therefore, future studies should focus on effects of different cytokine concentrations and combinations, which may have differential effects on neuronal integrity and survival.

Taken together, we show that distinct cytokines are a potential cause for neuronal damage in autoimmune CNS disease. In particular, synergistic cytokine exposure considerably impacts neuronal integrity. As compared to other studies, we could first demonstrate these effects in a human cell culture system and pinpoint the IL-17A/TNFα combination as most relevant neurodegenerative trigger. Future studies targeted at stopping neurodegeneration may consider controlling several cytokine pathways or simultaneous inhibition of combined cytokines.

## Supplementary Information

Below is the link to the electronic supplementary material.

Supplementary file1 (AVI 18140 KB)

Supplementary file2 (PDF 1658 KB)

## Data Availability

The datasets used and/or analysed during the current study are available from the corresponding author on reasonable request.

## Abbreviations

MS: Multiple sclerosis
CNS: Central nervous system
hNSC: Human neuronal stem cells
qPCR: Quantitative polymerase chain reaction
TNFα: Tumor necrosis factor
IFNγ: Interferon gamma
GM-CSF: Granulocyte-macrophage colony-stimulating factor (),
IL-17A: Interleukin 17A
IL-10: Interleukin 10
Th1: T helper cells type 1
Th17: T helper cells type 17
CSF: Cerebrospinal fluid
pwMS: Patients with MS
EAE: Experimental autoimmune encephalomyelitis
EGF: Epidermal growth factor
ß-FGF: Basic fibroblast growth factor
BDNF: Brain-derived neurotrophic factor
NT3: Neurotrophin 3
MAP2: microtubule-associated protein 2
TUBB3: Class III β-tubulin
RQ: Relative quantification
CHAT: Choline acetyltransferase
SLC6A: Solute carrier 6a
TH: Tyrosine hydroxylase
VGLUT1: Vesicular glutamate transporter
PBMC: Peripheral blood mononuclear cells
PwMS: Persons with Multiple Sclerosis
IL-17RA: Interleukin 17 receptor A
IL-17RC: Interleukin 17 receptor C
IL-10RA: Interleukin 10 receptor A
IL-10RB: Interleukin 10 receptor B
TNFRSF1A: Tumor necrosis factor receptor type 1A
TNFRSF1A: Tumor necrosis factor receptor type 1B
IFNGR1: Interferon gamma receptor 1
IFNGR2: Interferon gamma receptor 2
GRM1: Glutamate metabotropic receptor 1
GRIN1: Glutamate ionotropic receptor N-methyl-D-aspartate type subunit 1
GRIA1: Glutamate ionotropic receptor type α-amino-3-hydroxy-5-methylisoxazole-4 propionic acid subunit 1
