# Supplementary figures and images for: Inflammatory Cytokines Associated with Multiple Sclerosis Directly Induce Alterations of Neuronal Cytoarchitecture in Human Neurons
Source: J Neuroimmune Pharmacol. 2023 Mar 2;18(1-2):145–59. doi: 10.1007/s11481-023-10059-w (PMC10485132; doi:10.1007/s11481-023-10059-w)

Supplemental Figure 1

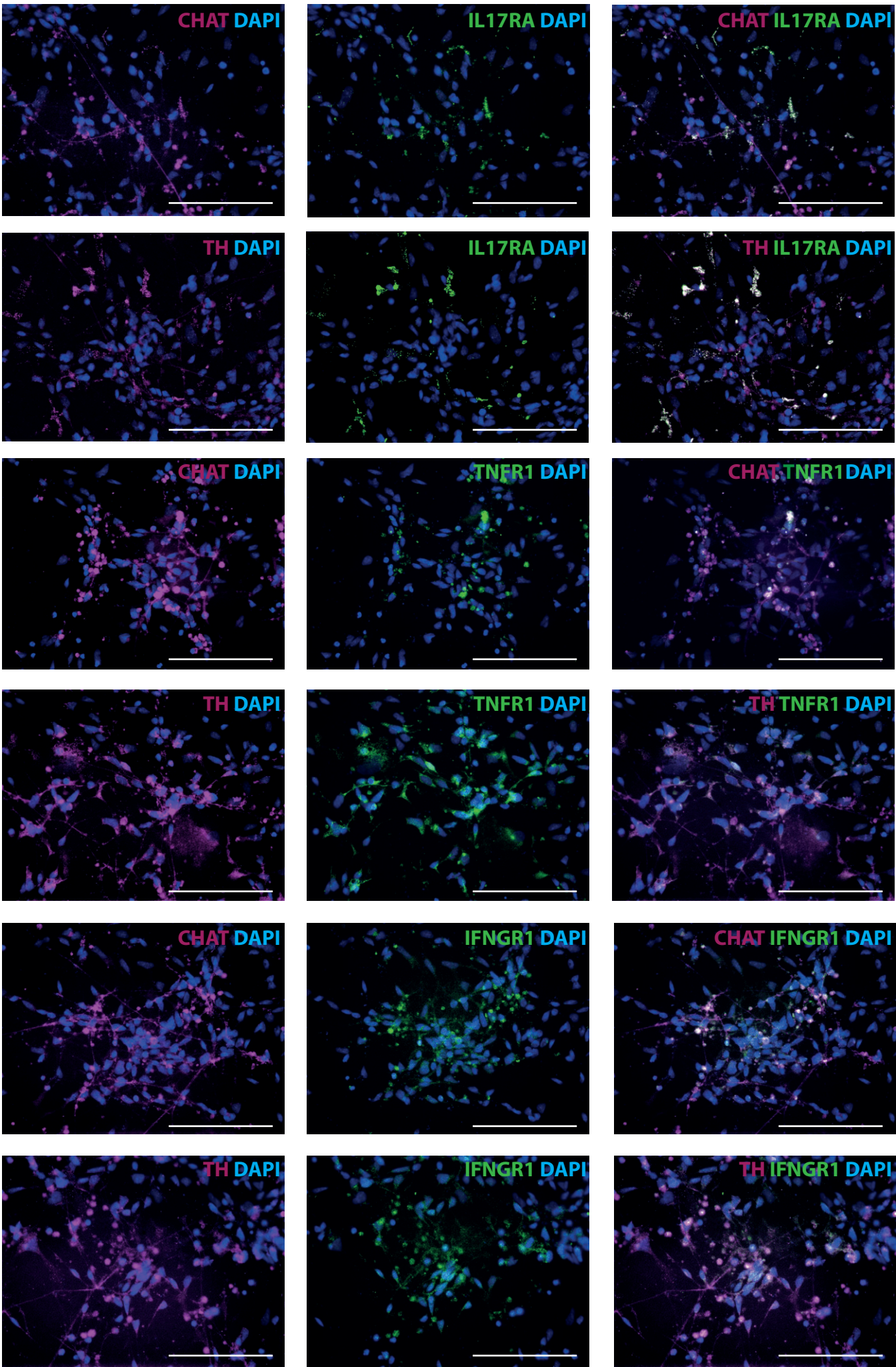

Supplemental Figure 2

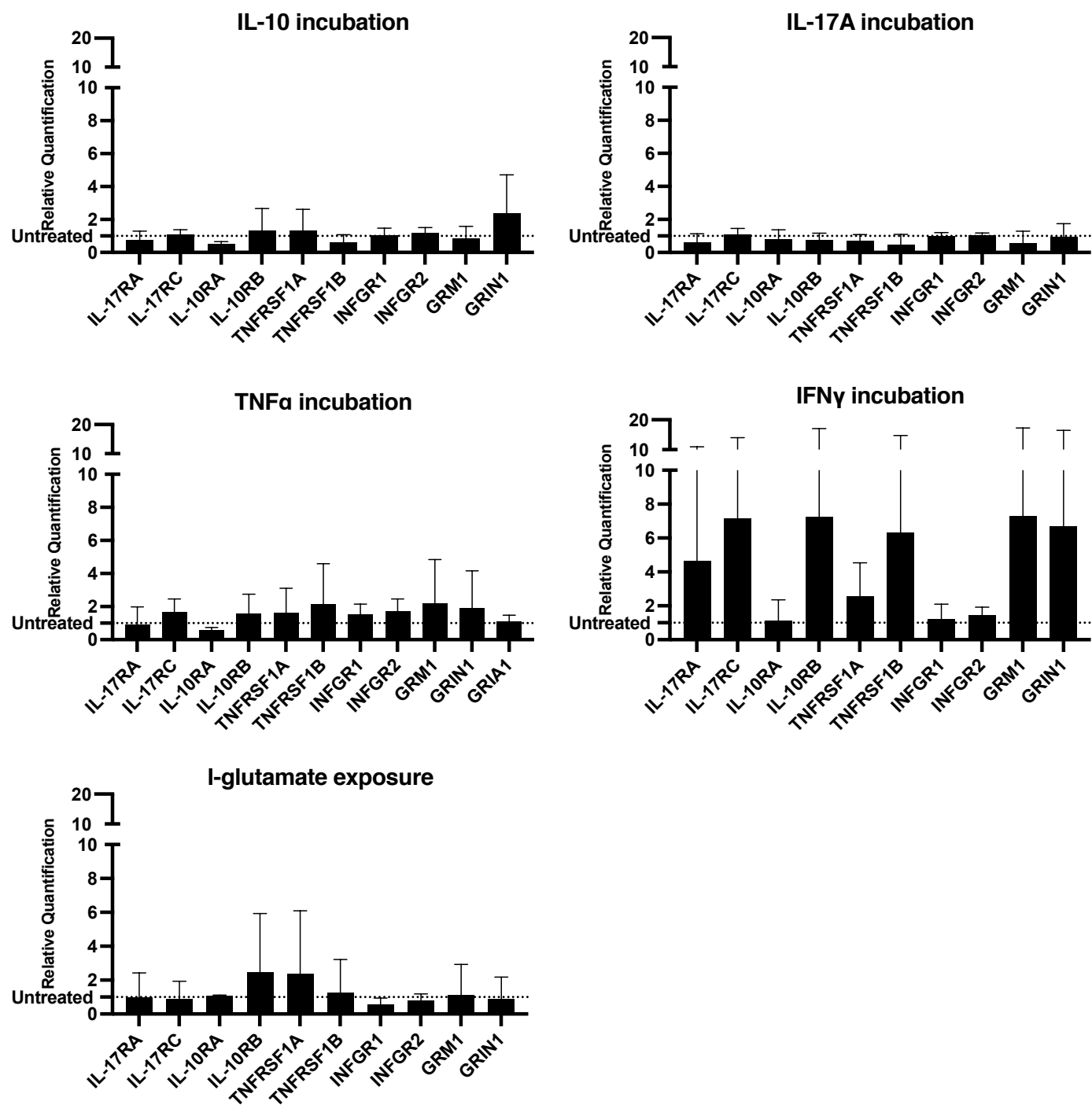

Supplement: Supplementary file 2 — Supplementary file2 (PDF 1658 KB) [file 11481_2023_10059_MOESM2_ESM.pdf]
